# Is the Cat–Owner Relationship Related to Cat–Wildlife Conflicts?

**DOI:** 10.3390/ani16172777

**Published:** 2026-09-03

**Authors:** Elke Schüttler, Natalia Rojas-Troncoso, Nicolás Berrios, Jaime E. Jiménez

**Affiliations:** 1 Centro Universitario Cabo de Hornos, Universidad de Magallanes, O’Higgins 310, Puerto Williams 6350000, Chile; 2 Cape Horn International Center (CHIC), O’Higgins 310, Puerto Williams 6350000, Chile; nrojast8@uc.cl; 3 Faculty of Agronomy and Natural Systems, Pontificia Universidad Católica de Chile, Av. Vicuña Mackenna 4860, Santiago de Chile 6904411, Chile; 4 Department of Ecology, Pontificia Universidad Católica de Chile, Av. Libertador Bernardo O’Higgins 340, Santiago de Chile 6904411, Chile; nberrios.bio@gmail.com; 5 Department of Biological Sciences and Advanced Environmental Research Institute (AERI), University of North Texas, 1155 Union Circle #305220, Denton, TX 76203-5017, USA; jaime.jimenez@unt.edu

**Keywords:** animal-borne cameras, cat–wildlife conflict, Chile, *Felis catus*, free-ranging cat, husbandry, hunting, individual variability, predation, sub-Antarctic ecosystems

## Abstract

Free-ranging pet cats have been identified as significant predators of wildlife. Managing this conflict is challenging, because control of movement ultimately lies with the owner. Previous research has focused on the influence of environmental, intrinsic, husbandry-, and personality-related factors on cats’ behavior when stepping out the door. However, an overlooked driver is the strength of the cat–owner relationship. In this explorative study, we used animal-borne video cameras to document the outdoor behavior of 12 cats and in parallel assessed the relationship with their owners through the Cat–Owner Relationship Scale (CORS), along with intrinsic (age and sex) and husbandry-related (cat’s role, cat’s restriction and roaming pattern, parasite treatment, and provisioning households) determinants. We found no statistical evidence of a direct influence of the cat–owner relationship on exploratory and hunting behaviors, but owners with higher CORS scores observed more harassment incidents by their cats and owners reporting more hunting behavior tended to own cats that roamed more in nature; those cats also explored more. We further observed high individual variability in hunting and time allocated to urban and natural environments. Comparing our results with the previous literature, we conclude that outdoor behavior largely varies between individual cats and is difficult to predict. However, the greater awareness of cat–wildlife conflict among owners with stronger bonds may provide an opportunity to promote changes in owner behavior.

## Simple Summary

Cats are major wildlife predators. Opportunities for owned cats to access the outdoors are partly shaped by owners’ housing and management decisions, although cats may subsequently move independently. Previous studies have investigated the factors that influence outdoor behavior, such as the environment, individual characteristics, care practices, and personality, whereas the cat–owner relationship has received little attention. Here, we attached video cameras to the collars of 12 cats to relate their outdoor behavior to the quality of the cat–owner relationship, along with their age, sex, and owner’s care practices. We did not detect evidence of a statistically significant association between whether cats explored or hunted and the degree of bonding, but owners indicating a closer relationship with their cats observed them disturbing wildlife more and owners reporting more hunting behavior tended to own cats that roamed in nature; those cats also explored more. Further, cats showed great variation in their hunting behaviors and where they spent time (urban or natural areas). We conclude that outdoor cats largely behave individually and that the cat–owner relationship alone did not explain outdoor behavior related to predation. However, the fact that owners with a stronger bond were more aware of cat–wildlife conflict has the potential to encourage changes in owner behavior.

## 1. Introduction

In the Anthropocene, the role of cats is no longer confined to that of companions and mousers in homes, gardens, and ships [1]. Domestic cats (*Felis catus*) are increasingly recognized as agents of biodiversity loss in numerous ecosystems around the globe (in Australia [2], in Canada [3], and in China [4]). Hundreds of millions of free-ranging pet and feral cats impact wildlife through predation. According to the synthesis of Lepczyk et al. [5] on global cat diet, cats are extreme generalist predators, preying up to 16.7% (347/2084) on species of conservation concern. Although cats predominantly consume birds, mammals, and reptiles upon availability (~90% [5]), they can also develop individual hunting preferences and specialize on scarce, vulnerable prey [6].

Indeed, cats are related to 63 species extinctions and—together with rodents—figure among the top mammalian invasive predators worldwide endangering birds [7]. But not only do cats hunt wildlife, they are also an overlooked risk for pathogen spillover to wildlife [8], can reduce the gene pool of wildcats through hybridization (e.g., the European wildcat *Felis silvestris* [9]), and compete with wild carnivores for food [10]. Cats may also indirectly reduce wildlife activity as a result of fear of their mere presence [11]. For example, bird fecundity can be diminished through a reduction in parental food load [12].

Due to the long-established relation of cats with humans, the management of free-ranging pet cats differs fundamentally from that of wild or feral carnivores. Because domestic cats are companion animals under human care, strategies to limit their outdoor access are often constrained by owners’ beliefs that cats should be free to roam and express their natural hunting behavior [13,14]. While these attitudes persist, a better understanding of the factors influencing cats’ behavior outdoors is needed to inform effective management strategies. Traditionally, these factors have been classified into environmental, intrinsic, and husbandry-related determinants.

Among the environmental predictors of cat outdoor behavior is the degree of urbanization. A lower residential density increased the home ranges of cats [15,16], but see [17]. Rural cat owners also reported higher frequencies of captured prey than urban owners, while the presence of trees significantly increased rates of predation on birds [18]. Regarding the intrinsic predictors of cat outdoor behavior, younger male cats have been found to have larger home ranges, as revealed by a meta-analysis involving 25 studies [15] and by GPS tracking of 875 cats from six countries [19]. The influence of sex was not always evident, though [16,20]. Husbandry-related drivers indicate a significant effect of intact cats [21] and intact males moving further [19], but this effect was not conclusive [15]. Providing high-protein food reduced the number of prey brought home by free-ranging cats [22]; similarly, cats fed with commercial or prepared food on a daily basis versus wheat bran or household scraps had fewer wild vertebrates in their scats [23]. Whether owners allowed full outdoor access or restricted cat movement during the night had a strong effect on 72 cats in Great Britain, with 75% larger home ranges in unrestricted cats [24], but see [16]. Finally, whether the household included children or whether dogs were present also increased free roaming in cats [25].

Although some of the forementioned drivers reflect the same tendency in the literature, there is still a considerable amount of variability at stake, reflected, for example, by “outlier cats” in home range studies [17,19]. Whether this remaining variability can be ascribed to personality traits in cats is an emerging field of research. For instance, high scores for extraversion (e.g., active, vigilant, curious traits) and low ones for neuroticism (e.g., insecure, anxious, fearful of people), two of the five personality traits in felids [26], were associated with more prey returned to home [24,27]. On the other hand, high levels of agreeableness (e.g., affectionate, friendly to people, gentle) and neuroticism were found in cats with smaller range sizes and less hunting propensity [28]. Despite these encouraging new studies, one systematically overlooked driver accounting for individual variability is the cat–owner relationship.

There is evidence that the quality of the cat–owner relationship may influence owners’ decisions regarding cat management. For example, owners who perceived their cat as a “child” or “best friend,” rather than simply as a “pet,” were less likely to provide outdoor access [29]. Similarly, owners characterized by a “co-dependent” relationship, shaped by frequent cat–owner interactions (e.g., the owner typically remains with the cat during feeding), would not allow their cats outdoor access, in contrast to “casual” cat–owner relationships, in which cats seek less proximity to their owners [30]. Another indicator of the cat–owner relationship is the frequency of play between cats and their owners [31,32]. Notably, Cecchetti et al. [22] found that only 5–10 min of object play with cats reduced prey brought home by 25%. This finding is consistent with the experimental study by Hall and Bradshaw [33], who demonstrated that object play and predatory behavior are motivationally linked, suggesting that object play may partially satisfy cats’ motivation to hunt. Building on this evidence, we aimed to investigate whether the overall cat–owner relationship is associated with cats’ outdoor behavior, as has recently been described for dogs [34].

To document cat behavior, we decided to use animal-borne cameras, an outstanding technique, which has brought new insights into the hunting behavior of cats since Loyd et al. [35] published their landmark study in 2013. This study demonstrated that estimates of prey capture based on prey brought home substantially underestimate cats’ successful hunting, as evidenced by video recordings [35]. Thereafter, animal-borne video cameras have not only been used to document predation itself but also to investigate the determinants of predation. Thus, camera data showed that feral cats in Australia were much better hunters in open versus dense habitat [36]. Similarly, the hunting frequency of pet cats in France depended on owner-ascribed personality traits [28]. Cat-mounted cameras even documented human behaviors potentially associated with parasite transmission in cats in Chad, Central Africa [37], highlighting the diverse research areas in which this method can be applied. To our knowledge, our study uses animal-borne cameras for the first time in Chile, a country with highly biodiverse regions where free-ranging cats are receiving increasing scientific attention, e.g., regarding their movement ecology [38] and owner willingness to feed insect meals [39]. Specifically, we worked on a near-pristine island that lacks native terrestrial predators [40], a particularly challenging setting for cat–wildlife conflict [5,41].

In this study, we explicitly asked (1) which behaviors occur in outdoor cats in southernmost Chile, (2) where on an anthropogenic–natural environmental gradient do cats spend more time, and (3) which factors (intrinsic, husbandry-related, and cat–owner relationship) determine behaviors related to exploration and hunting behaviors in outdoor cats. We predicted that cats of owners indicating a stronger relationship with their cats would get less engaged in cat–wildlife conflict, exhibiting less exploratory and hunting behavior (playing together decreases predation rates [22]). Those behaviors would also be less present in older cats and female cats without dependent kittens (male and younger cats roam further [15,19]) and in cats whose owners provide higher levels of care and surveillance (commercial food decreases the consumption of wild prey [23], and restricted cats roam less [24]). We endeavor to draw more attention to the role owners play in shaping their cats’ outdoor behavior through their interactions and emotional bonds. In this way, an additional dimension could be incorporated into cat management campaigns, namely, the promotion of a strong cat–owner relationship. Such campaigns are particularly relevant wherever cats range near wild habitats.

## 2. Materials and Methods

### 2.1. Study Area

**Figure 1:**
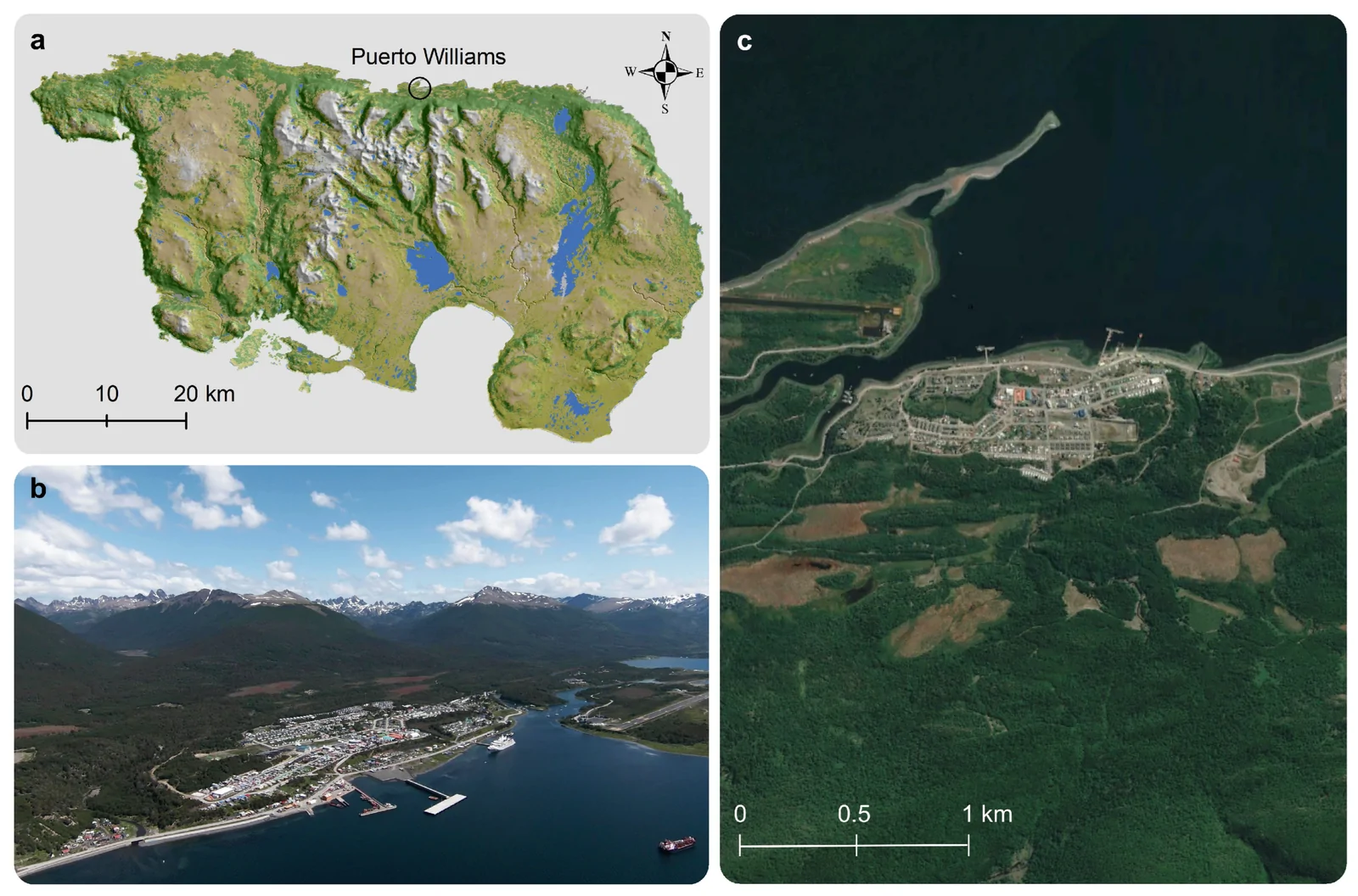
Study area for 12 free-ranging cats on Navarino Island (**a**), southern Chile. The only major town is Puerto Williams (**b**), a small town located in near-pristine nature (**c**). Photograph: courtesy of Matías Troncoso, satellite map: Google Earth (2025).

We worked on Navarino Island (2.528 km^2^), southernmost Chile (55° S). Navarino Island is part of the Cape Horn Biosphere Reserve, outstanding for its near-pristine character of sub-Antarctic landscapes composed of southern beech forests (*Nothofagus* spp.), Magellanic tundra (*Sphagnum* spp.), high-Andean habitats, lakes, and rivers. The only larger town on the island is Puerto Williams, with approximately 2.200 inhabitants (Figure 1). Additionally, several small-scale farms are located along the northern coast of the island, and there is a small fishing town in the east of the island. Introduced predators are American mink (*Neogale vison*), domestic dogs (*Canis familiaris*), and domestic cats. Some free-ranging cats have been reported by their owners to disappear for up to seven days, and cats also get lost (12 cats over a 10-year period) [42]. In general, there are more dogs than cats kept as pets in Puerto Williams (40% versus 14% of 215 households), and most cats are of local origin (67%, *n* = 30) [42].

### 2.2. Participating Cats

A total of 12 domestic cats participated in our study (December–April 2017/18 and 2018/19). Cats were recruited by inviting cat owners known by local researchers and veterinarians to be possibly interested and by approaching households with cats in their gardens. Only healthy cats, checked by a veterinarian during an in situ visit, adult cats aged ≥1 years, and cats that spent at least two hours outside each day were selected for the study. Cats showing signs of stress when wearing the camera device during their first session were excluded from the study (*n* = 3, from 15 selected cats). Ten participating cats were from households in Puerto Williams, while two cats lived outside the town.

### 2.3. Cat–Owner Relationship Scale

We used the Cat–Owner Relationship Scale (CORS) developed by Howell et al. [31] to assess the strength of the relationship between cats and their owners, from the owner’s perspective (see Table S1 of the Supplementary Materials for a translated (into Spanish) slightly modified version of the original CORS). The scale is an adaptation of the widely used Monash Dog–Owner Relationship Scale [43] to cats and is divided into three subscales: (1) The Pet–Owner Interaction subscale (in short: Interaction) evaluates how often the owner engages in activities such as playing, petting, or talking with the cat; (2) the Perceived Emotional Closeness subscale (in short: Emotion) evaluates the extent to which the owner agrees with statements such as “My pet helps me get through tough times”; and (3) the Perceived Costs subscale (in short: Costs) evaluates how strongly the owner feels the burden of having a cat. We followed the scoring instructions in Howell et al. [31] and calculated a score from 1 to 5 for each subscale; the higher the score, the better the quality of the perceived relationship, meaning frequent interaction, high levels of emotional closeness, and low levels of burden. We used Cronbach’s alpha to determine the internal consistency of each subscale, using a value of >0.7 as an acceptable level of consistency.

### 2.4. Demographic Cat–Owner Data

We also applied a questionnaire to collect background data on cats and owners, adapted from previous research in the study area [42]. This questionnaire contained 20 closed- and opened-ended questions on the cats’ background, husbandry, restriction of movement, and observed cat–wildlife interactions, as well as personal information of owners (Table A1).

### 2.5. Animal-Borne Cameras

**Figure 2:**
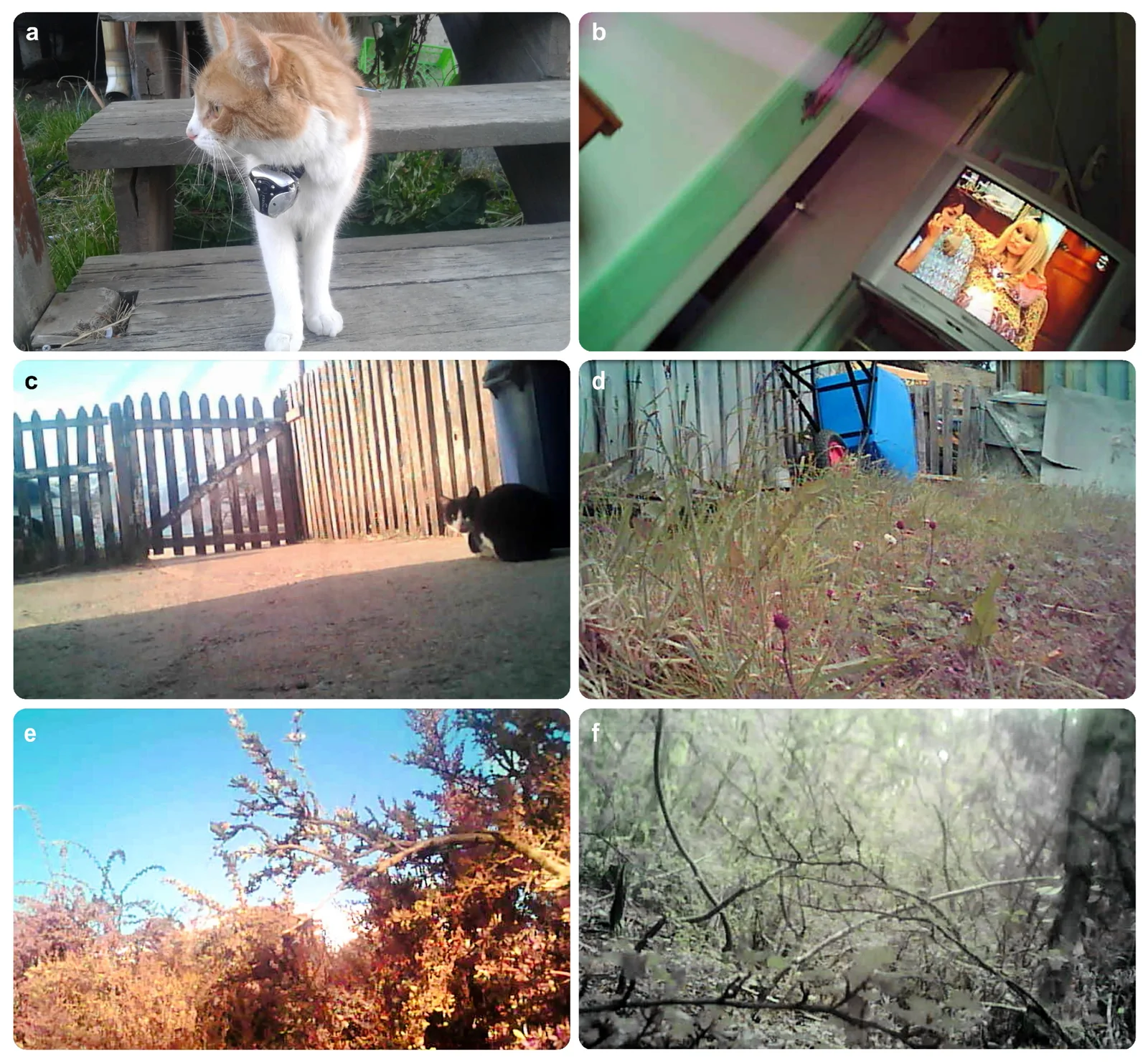
Video screenshots taken by animal-borne cameras (**a**) worn by 12 free-ranging cats on Navarino Island, southern Chile. The screenshots show examples of cats inside a house (**b**), in a grey urban environment (**c**), in a green urban environment (**d**), and in natural habitats such as shrubland (**e**) and forest (**f**).

We fitted 12 cats with small video cameras (in what follows, videocams) designed for domestic cats (Eyenimal; NUM’AXES company, Olivet, France) attached to commercial cat collars (Figure 2a). Monitoring took place during two summers (total effort in Table 1). The videocam and collar weighed ~40 g, which is <1% of the average body weight of a domestic cat. It recorded sound and was equipped with infrared light to film during dark hours. We aimed at recording a minimum of 3 h per cat. As the batteries of the videocam lasted a maximum of ~3 h once fully charged, we estimated a minimum effort of two sessions or visits to the cat owner. The sessions took place at different times of the day, 6 AM being the start of the earliest morning session and 1 AM that of the latest night session. However, scheduling ultimately depended on the availability of the owner and the outdoor schedule of cats. Note that cats could enter the home (or another house in the vicinity) again after starting the video session outside. Ideally, the monitoring of each cat was completed within a period of 4 days to 2 weeks. Each session lasted on average 2.05 ± 0.52 h (range: 0.09–5.33, *n* = 52). As we were interested in cat–wildlife interactions, the device was used in the mode to start recording when movement was detected. A blue light shone when cameras were working, which might have had an influence on cat–animal interactions.

### 2.6. Analysis of Cat Behaviors

For our video analysis we used the “catcam ethogram” of Huck & Watson [44], who adapted the detailed “standardized ethogram for the Felidae” by Stanton et al. [45] to cat-camera footage. Of the 36 behaviors in the catcam ethogram, 25 behaviors occurred in our video footage. Additionally, we recorded the behaviors purr, mentioned in [45], and interaction with subjects or objects (own classification), totaling 28 behaviors (detailed description of our ethogram in Table A2). Depending on the duration of each behavior, we recorded short instantaneous actions as frequencies of occurrence (i.e., point state behaviors) and longer actions or postures as both frequencies of occurrence and time the behavior persisted (i.e., state event behaviors). Three exemplary video excerpts are provided in the Supplementary Materials (Videos S1–S3). For video coding, we used the open-source software BORIS, version 7.9.4 [46].

To familiarize ourselves with the catcam ethogram and establish consistent coding procedures, an internship student initially coded five cats (F02, F03, F05, M01, and M05), while E.S. coded three cats (F01, M03, and M05). Coding procedures and uncertainties were subsequently discussed until consensus was reached. Based on this process, the final codifier (N.R.T.) was trained, and one cat was coded independently by N.R.T. and E.S. to finish the calibration. However, we did not assess formal interobserver reliability, which is a limitation of the study.

To facilitate statistical analyses, we condensed individual behaviors into broader behavioral categories according to their shared functional roles. For this purpose, we followed the categorization of Stanton et al. [45], who suggested 15 common behavioral categories adapted from the literature. Our behaviors fell into 13 of Stanton et al.’s categories; the exceptions were aggressive and stereotypic behaviors, which were not observed among our participating cats. The category exploratory behaviors, for instance, is defined by investigative behaviors related to the exploration of the environment or the investigation of specific stimuli. The category feeding refers to any behavior related to the acquisition and consumption of food. Each behavior could occur in several categories. Overall, most behaviors fell into the category active, followed by the categories calm, feeding, locomotion, inactive, and maintenance (Table A2).

Additionally, we summarized the time cats spent (i) inside a house (the owner’s home or another home) and in the urban environment, differentiating between (ii) grey urban, meaning lacking predominant vegetation, e.g., under a house, in a shed, on the street, or in a backyard, and (iii) green urban environments, meaning gardens and public green spaces, as well as natural habitats such as (iv) shrubland and (v) forest (Figure 2b–f). The mean distance of the cat’s home to the limit of the urban area of Puerto Williams, where native forest could be accessed, was 80 ± 122.9 m (median: 35, range: 5–450); therefore, we supposed that cats could have visited a natural environment independent of their home locations.

### 2.7. Statistical Analysis

We used box plots to summarize the cats’ overall behaviors across categories. To check whether hunting-related (i.e., foraging, hunting, stalking, and stare—hunting) and exploratory-related behaviors (exploring, interaction—objects, interaction—social, and watching) showed significant differences between individuals, we performed Kruskal–Wallis tests. The percentage of time spent in each of the five above-mentioned environments (i.e., inside, grey urban, green urban, shrubland, and forest environments) was drawn in bar plots for each cat. Complementarily, we performed a Principal Component Analysis (PCA) to reveal whether cats used these five environments individually or eventually could be grouped (e.g., by their sex). Since each cat’s environmental space use was represented by proportions that together totaled 100% (i.e., an increase in one habitat automatically means a decrease in another), these variables were not independent. To be able to meet the PCA assumption of independency, we therefore applied a centered log-ratio transformation to make the data suitable for PCA.

To understand which factors might influence the cat’s outdoor behavior, we used non-parametric univariate tests in all combinations (*n* = 40) for the five response variables (i.e., owner observation of hunting/harassment, hunting- and exploratory-related behaviors, and time spent in nature, as recorded by videocams) and eight explanatory variables (i.e., biological cat characteristics, husbandry-related characteristics, and the cat–owner relationship questionnaire; description in Table 2). Additionally, we checked whether the response variables were related to one another (10 combinations). To test whether there were relationships between two categorical variables, we used Fisher exact tests (*n* = 11); for the comparison of distributions between a continuous and a categorical variable, we used Wilcoxon rank sum tests for independent samples (*n* = 27); and to assess the strength of the relationship between two continuous variables, we used Spearman correlations (*n* = 12). We corrected multiple comparisons using the Holm–Bonferroni procedure within each family of response variables, correcting tests across the eight explanatory variables. We also corrected the ten tests among the response variables themselves. Given our low sample size (12 individuals), inferential tests may have limited the power to detect underlying trends in the data. Therefore, we computed effect sizes as rank–biserial correlations, obtained from the Wilcoxon Z statistic (r=Z/√N), and report their 95% confidence intervals to provide an estimate of the magnitude and uncertainty of the observed effects for visually detected trends. A large effect between groups is given with |rc| ≥ 0.43 (range: −1 to 1 [47]). All statistical analyses were carried out in the program R, version 4.1.2 [48]. We also declare the use of ChatGPT (version GPT-5.5, OpenAI, San Francisco, CA, USA) for orientation in R programming related to the PCA, the Holm–Bonferroni procedure, and the choice of effect sizes. All generated code was verified independently. Rank–biserial correlations were calculated using the R package “effectsize” [49]. Data are available as an Excel file in Table S2 of the Supplementary Materials.

## 3. Results

### 3.1. Cat Background

Half of the 12 participating cats were female, half were male; the mean age was 3.7 ± 3.1 years (range: 1–13). The majority of the cats (75%, 9/12) were born on Navarino Island, and most (83.3%, 10/12) were held for companionship. The husbandry standard of the participating cats was relatively high. A total of 91.7% (11/12) of the cats were neutered; 83.3% (10/12) were vaccinated against rabies within the last year; and 75% (9/12) were regularly treated against parasites, but six of them were due, i.e., more than 3 months had passed since the last medical treatment. Cats were typically fed with dry food (91.7%, 11/12), and only two cats ate at more than one household.

According to their owners, only two out of the 12 cats were restricted most of the time, two cats entered their homes at night, and the remaining cats (66.7%) were able to roam freely day and night. Regarding the cat’s reported outdoor behavior, 66.7% (8/12) of the cats had brought home animals; the majority brought birds (7/12) and mice (5/12). Species mentioned were the Austral Parakeet (*Enicognathus ferrugineus*) and the Rufous-collared Sparrow (*Zonotrichia capensis*). However, owners observed most of their cats hunting (83.3%, 10/12). Again, birds were among the animals most frequently harassed (seven out of 12 cats), followed by mice (4/12), as well as mink (1/12) and flies (1/12). Two participants mentioned that their cats had brought home rabbits and hunted lizards. Finally, five out of the 12 cats (41.7%) had gone lost for spans between 1 and 30 days (7.8 ± 12.4).

### 3.2. Cat–Owner Background

Cat owners were 67% women and 33% men (*n* = 12); half of them had a university degree, 25% had finished high school, 16.7% had finished elementary school, and 0.8% had completed technical training. The participants’ mean age was 42.5 ± 14.7 years (range: 26–78), and their mean residence time on Navarino Island was 12.1 ± 15 years (range: 2.5–45). In total, the participants owed 1.2 ± 0.4 cats (range: 1–2) and 0.7 ± 1 dogs (range: 0–3).

### 3.3. Cat–Owner Relationship

Cronbach’s α was 0.60 for the Interaction subscale of the CORS, 0.84 for the Emotion subscale, and 0.61 for the Cost subscale. Dropping question 26 from the Interaction subscale raised α to an acceptable level of 0.74. As the Cost subscale lacked internal consistency, we deleted it from the analysis. All participants expressed high levels of interaction with their cats (mean = 4.7 ± 0.5, range: 3.4–5; scale scores were 1–5) and a moderate emotional bond was reflected by the questionnaires (mean = 3.8 ± 0.7, range: 2.4–4.6; scale scores were 1–5).

### 3.4. Cat Outdoor Behavior

**Figure 3:**
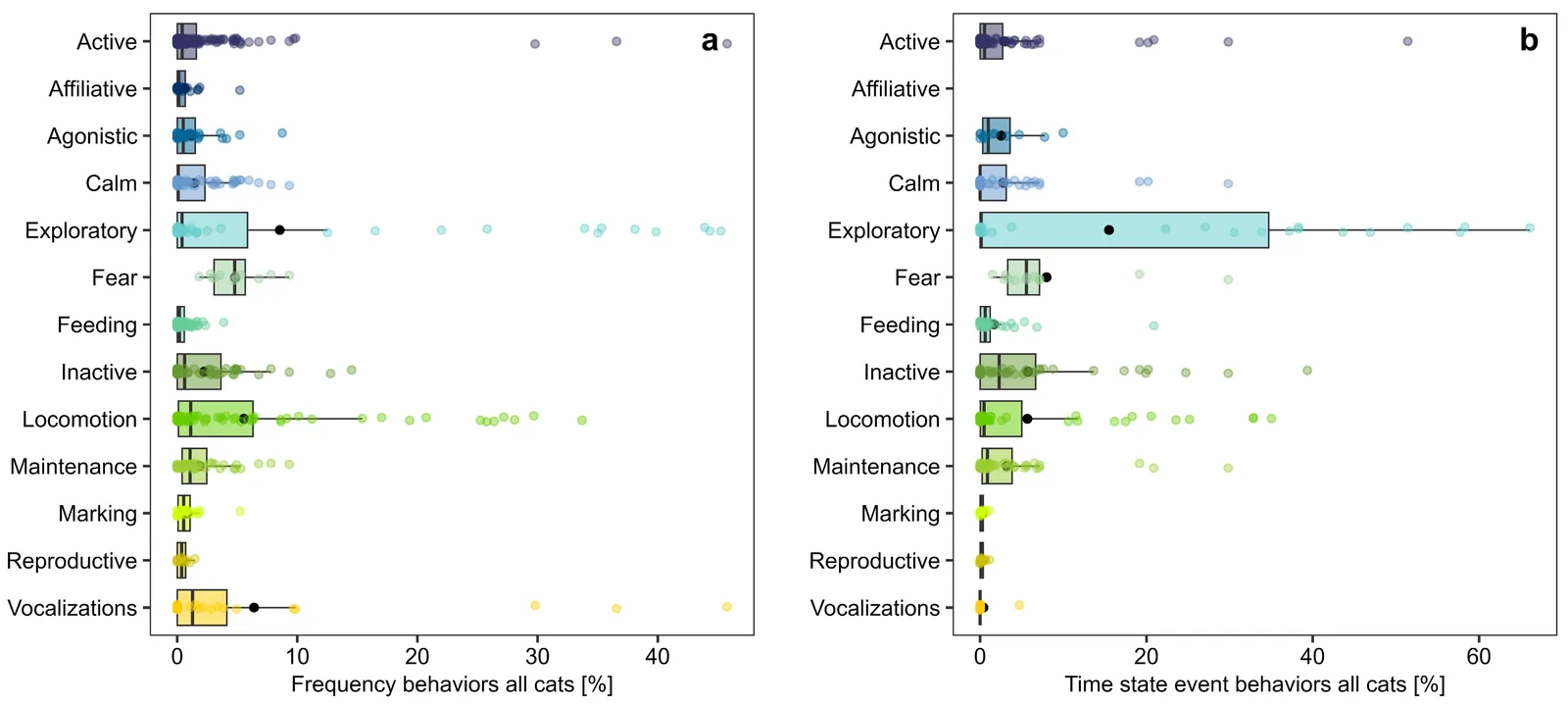
Box plots of percentages of frequencies (**a**) and time (only state event behaviors) (**b**) for 13 categories of behaviors summarized over 12 free-ranging cats on Navarino Island, southern Chile. Black dots represent the mean values for each category. Each category is composed of several behaviors; behaviors can occur in several categories (Table A2). Note that the category fear is only composed of the behavior grooming, as other fear-related behaviors were not observed; the category affiliative only had point event behaviors; therefore, we miss values in (**b**).

Cats were monitored for a total of 106 h and 24 min, from which we analyzed a total of 50 h and 33 min of camera footage during which cats were active and cameras were recording. Because cats differed in both the total number of observed behaviors and the total time spent performing behaviors, all data were expressed as percentages, using each cat’s total observed behaviors for frequencies and total observation time for durations. The most often displayed behaviors (frequencies of all behaviors across all cats, Figure 3a) were related to exploratory behaviors, including exploring, interaction—objects, interaction—social, and watching (mean = 8.5 ± 15.1, median = 0.4, range: 0–45.3). The category vocalizations, representing the behaviors purring and vocalizing, was the second most displayed behavior (mean = 6.4 ± 12.5, median = 1.3, range: 0–45.8); locomotion (approaching, climbing, jumping, running, stalking, and walking behaviors) occupied the third place (mean = 5.5 ± 8.8, median = 1.1, range: 0–33.7). The category feeding, together with the behaviors drinking, eating, foraging, hunting, stalking, and stare—hunting, was relatively little displayed among cats (mean = 0.5 ± 0.7, median = 0.2, range: 0–3.9). Regarding time spent in the different behavioral categories (only state event behaviors across all cats, Figure 3b), cats also occupied most time in exploratory behaviors (mean = 15.5 ± 21.9, median = 0.1, range: 0–66.1), followed by fear (only behavior grooming; mean = 8.0 ± 8.2, median = 5.6, range: 1.5–29.8) and inactive behaviors (grooming, lying, resting alert, sleeping, stretching behaviors; mean = 5.7 ± 8.8, median = 2.3, range: 1.5–39.4).

**Figure 4:**
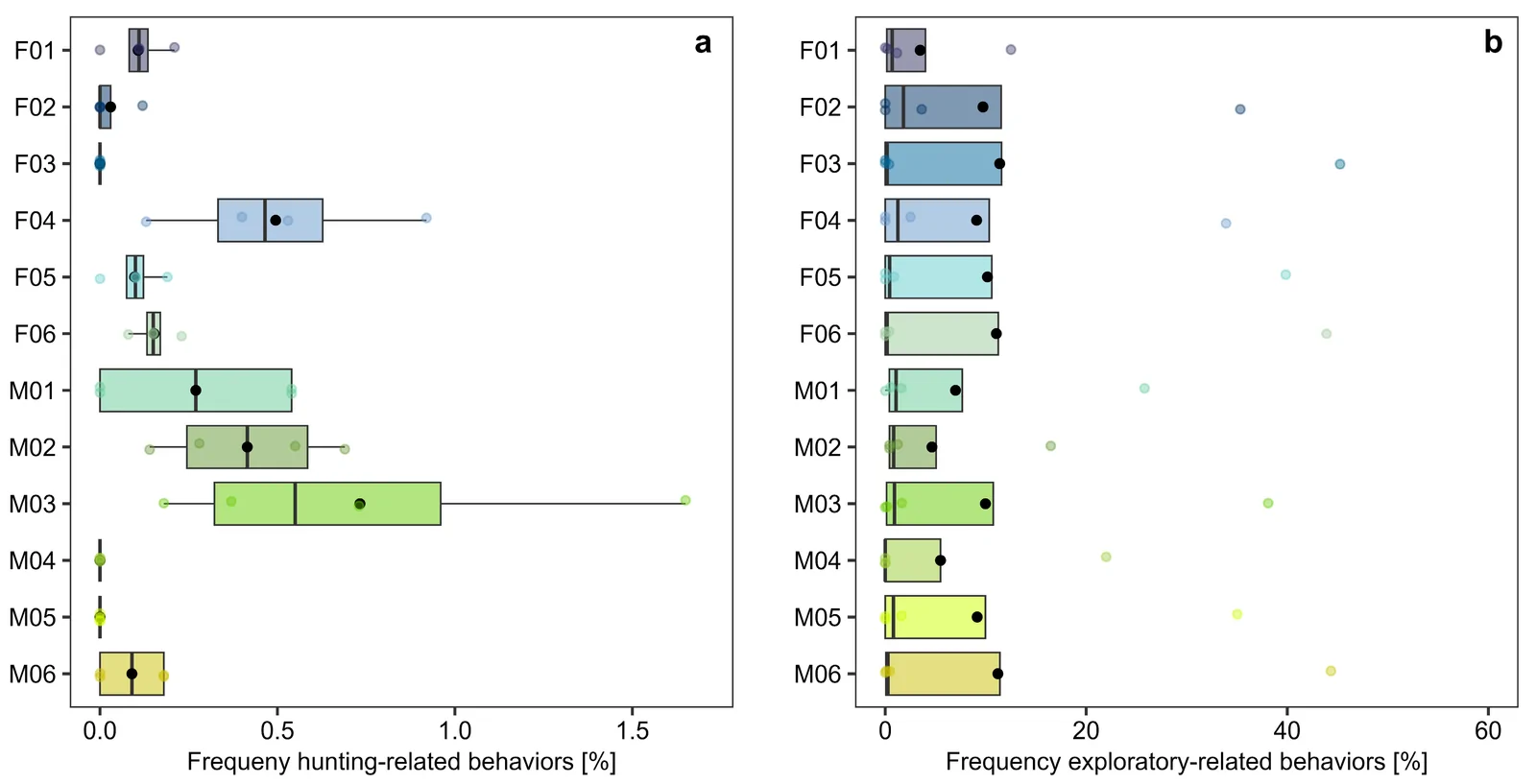
Box plots of percentages of frequencies for hunting-related behaviors (**a**) (foraging, hunting, stalking, and stare—hunting) and exploratory-related behaviors (**b**) (exploring, interaction—objects, interaction—social, and watching) for 12 free-ranging cats on Navarino Island, southern Chile. Black dots represent the mean values for each cat.

Regarding the hunting-related behaviors (foraging, hunting, stalking, and stare—hunting) the Kruskal–Wallis test was significant (H(11) = 30.96, *p* = 0.001), indicating high individual differences between individuals (Figure 4a). A rural cat (M03) showed the greatest values for hunting (mean = 0.73 ± 0.65, median = 0.55, range: 0.18–1.7). No successful hunting event could be filmed; only sniffing by M06 of an already dead bird in the backyard, identified as a Rufous-collared Sparrow, was observed (Video S1 of the Supplementary Materials). However, the exploratory-related behaviors (i.e., exploring, interaction—objects, interaction—social, and watching behaviors) yielded no significant differences between individuals (H(11) = 2.41, *p* = 1.0; Figure 4b).

**Figure 5:**
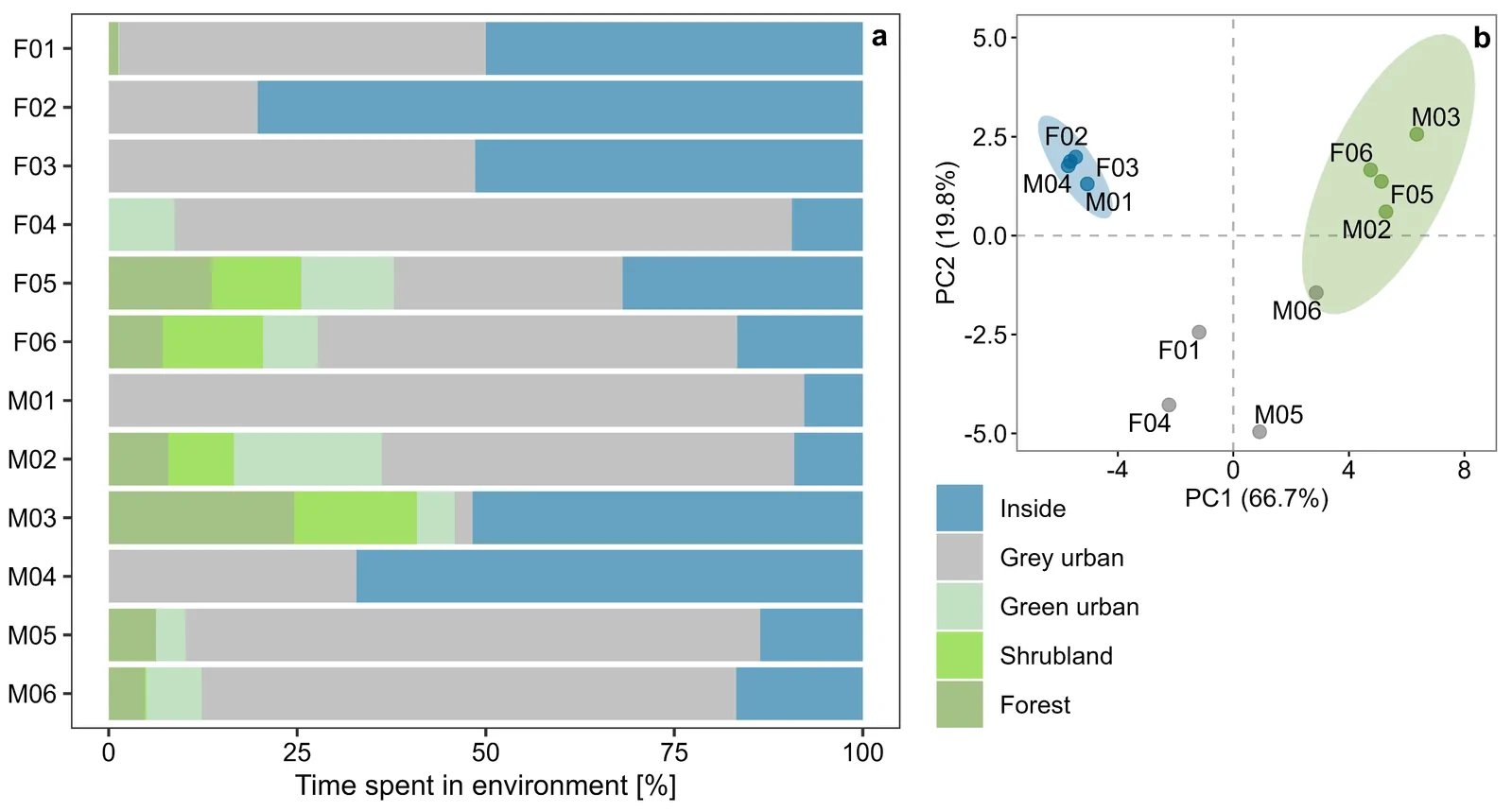
Bar plots of percentages of time (**a**) spent inside a house, in urban areas without vegetation (i.e., grey urban areas), in green urban areas such as gardens (i.e., green urban areas), in shrubland, and in forest habitats for 12 free-ranging cats on Navarino Island, southern Chile. Principal Component Analysis (PCA) plot (**b**) showing two clusters separating individuals with no time allocation in natural habitats (blue dots) and individuals spending time in all types of environmental space (green dots), while four individuals occupied an intermediate position (grey dots). Note that F05 and M03 were rural cats with immediate natural habitats in their surroundings.

Cats had individual preferences in regard to which environment they spent their time in (Figure 5a). Overall, they spent most time in urban areas without vegetation (grey urban) (mean = 2:14 ± 1:40 hh:min, median = 2:21, range: 0:08–5:44), that is, between 2.4 and 92.2% of the total time they were monitored. Cats spent also much time inside a house (mean = 1:3 ± 1:13 hh:min, median = 1:21, range: 0:04–3:11; i.e., 7.8–80.2% of total time monitored). Whether cats frequented natural habitats (i.e., shrubland and forest) was individually different (mean = 0:37 ± 0:55 hh:min, median = 0:08, range: 0:00–2:25; i.e., 0.0–40.9% of total time monitored). The first two components of the PCA together explained 86.5% of the variance and revealed two main clusters of individuals in the ordination space (Figure 5b): Four individuals (F02, F03, M01, and M04) did not frequent natural areas, while another four cats (M02, M03, F05, and F06) used all five types of environments. The rest of the individuals (F01, F04, M05, and M06) were positioned outside these clusters, indicating intermediate behavioral profiles.

### 3.5. Predictors of Cat Outdoor Behavior

**Figure 6:**
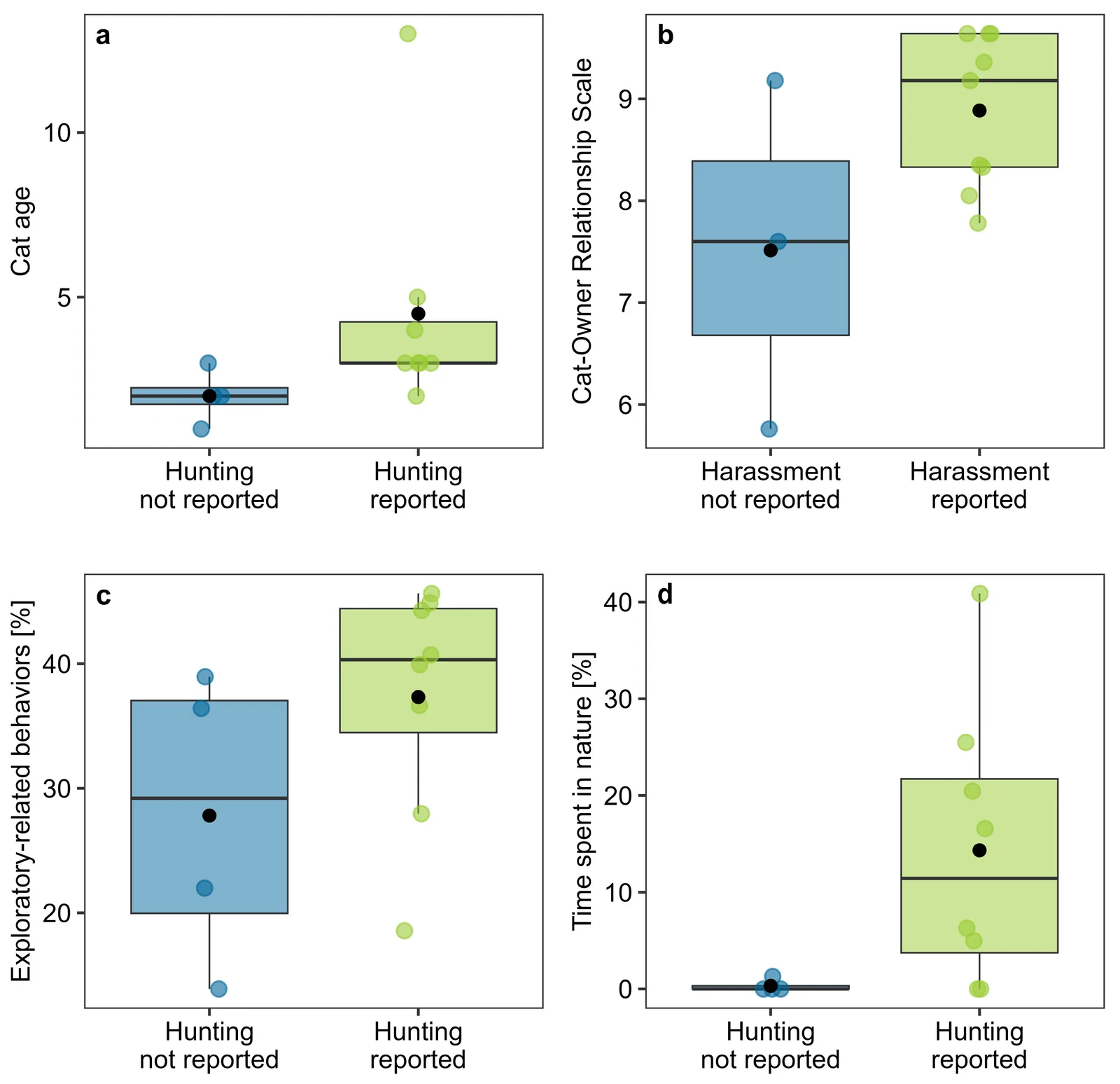
Box plots of group effects with large effect sizes following Wilcoxon rank sum tests for intrinsic and husbandry-related factors, and the cat–owner relationship for 12 free-ranging cats on Navarino Island, southern Chile: (**a**) cat age versus owner-reported hunting (r_rb_ = −0.75), (**b**) Cat–Owner Relationship Scale versus owner-reported harassment (r_rb_ = −0.67), (**c**) exploratory behaviors versus owner-reported hunting (r_rb_ = −0.62), and (**d**) cat’s time spent in nature versus owner-reported hunting (r_rb_ = −0.69). Black dots represent the mean values for each category.

Of the 40 non-parametric tests (Fisher exact tests, Wilcoxon rank sum tests, and Spearman correlations) of response and explanatory variables (Table 2), no test yielded a significant relationship after the Holm–Bonferroni correction; however, we detected three visual trends with large effect sizes [47]. Older cats were reported to hunt more than younger cats (U = 4, *p* = 0.328; r_rb_ = −0.75, 95% CI: [−0.93, −0.25]; Figure 6a). Owners with higher CORS loadings seemingly observed their cats’ harassing birds or mammals more than owners with lower loadings (U = 4.5, *p* = 0.904; r_rb_ = −0.67, 95% CI: [−0.92, −0.02]; Figure 6b), and cats reported by their owners to engage in hunting showed higher levels of exploratory-related behaviors (U = 6, *p* = 0.981; r_rb_ = −0.62, 95% CI: [−0.90, −0.01]; Figure 6c).

Regarding the relationships between the five response variables themselves, none of the 10 non-parametric tests was significant. Nevertheless, we detected a trend that cats that spent more time in natural habitats than in urban habitats or inside homes were observed by their owners to hunt more (U = 5, *p* = 0.64; r_rb_ = −0.69, 95% CI: [−0.92, −0.12]; Figure 6d). Those cats (spending more time in nature) also showed higher percentages of hunting-related behaviors; these two variables were moderately correlated, r_s_ (10) = 0.37 (*p* = 1.0).

## 4. Discussion

This study documented the detailed outdoor behavior of 12 pet cats in a small town surrounded by near-pristine sub-Antarctic ecosystems in southern Chile. Our main finding indicates that cats’ behaviors related to cat–wildlife interactions are more likely the result of individual variability than driven by intrinsic, husbandry-related factors or the strength of the cat–owner relationship. Importantly, no active predation event was recorded during 50.5 h of monitoring. Therefore, the exploratory and hunting behaviors can only be interpreted within the framework of the landscape of fear [11,12].

Different to our predictions, we found no evidence of a negative correlation between the cat–owner relationship and the cats’ video-evidenced exploration and hunting behaviors; neither did intrinsic or husbandry-related factors matter. However, we detected two trends. On the one hand, owners with higher scores on the Cat–Owner Relationship Scale observed more harassment incidents in their cats; on the other hand, cats whose owners observed them hunting more allocated more time to natural than to urban environments. This is an interesting finding, as increased attention of owners toward their cats—the product of a closer relationship—raised their awareness of cat–wildlife conflict. Indeed, the recognition of the cat as an undesired predator should incentivize some owners to assume responsibility for managing their cats’ behavior [50]. For example, cat owners significantly changed their answer options for the statement “Domestic cats killing wildlife is a serious problem” from “disagree” to “agree” after documenting their cat’s prey in the framework of a 4-week participatory Citizen Science project [51]. However, broader evidence suggests that hunting is widely regarded as a natural behavior in cats [50] and that concern about cats’ impacts on wildlife do not influence owners’ decisions to restrict their cats’ movements [14,52], whereas concerns about cats’ safety do [25,53,54]. Notably, concern for a cat’s safety is reflected in the CORS, which includes the item “How traumatic do you think it will be for you when your cat dies?” [31]. Consistent with this, owners with stronger emotional attachments to their cats have been shown to be more likely to restrict their cats’ outdoor access than less attached owners [29,30]. Therefore, cat containment campaigns may be more effective if they emphasize messages related to cats’ safety and the owner–cat bond, particularly when concern for wildlife is insufficient to motivate behavior change [54].

In this explorative study, we highlighted the cat–owner relationship as a driver of cat–wildlife conflict, but further research is needed. Such research could investigate the influence of the CORS [31] on home ranges of free-ranging cats or the strength of the cat–owner bond [30] on prey caught by cats. Importantly, the cat’s perspective on the bond might not necessarily reflect a questionnaire-based evaluation of the owner. Therefore, experimental assessments like the Strange Situation Test [55] would be an ideal additional test. This test originally evaluated attachment between infants and their mothers through the recording of exploratory, proximity-seeking, or contact-maintenance behaviors in one-year-olds in the presence and absence of a stranger and the mother and has been adapted to pet–owner dyads to evaluate bonding in a stressful situation (see [34] for dogs and [56,57] for cats). Definitely, a considerable number of individuals should be tested, as the cat–wildlife literature points to fairly idiosyncratic behavior in free-ranging cats (e.g., [6,28]).

Individual variability in the frequency of hunting-related behaviors was indeed a highly significant result in our study (Figure 4a). Reviewed by Cecchetti et al. [58], differences in the hunting behavior of cats can be ascribed to individual nutrient requirements driven by the type of provisioned food [22], but also the feeding scheme (here, not assessed). For example, free access to food ingested as several small daily meals might simulate feeding patterns in the wild [59], thereby reducing the frequency of hunting [22]. The early life experience (e.g., teaching by the mother) does also form individualistic hunting behaviors [58]. Recently, certain personality traits have been linked to hunting activity, with active, curious, inquisitive cats scoring high for the trait cluster extraversion [27,28].

A cat’s preference for its environment appears to be individually determined as well. Although we found two PCA clusters of cats preferring human-made surroundings versus cats spending their time along the gradient of human-made to more natural environments, other cats did not fit into these two clusters (Figure 5). Cats are described to have home ranges closer to 1.5 ha (mean kernel density estimate [60]) than to the upper range of 10.9 ha [61], staying normally as close as 60–100 m to their homes [16,62]. Given these movement ranges, the four cats that selected homes and urban areas in our study should have had access to forested habitat, as their homes were located only 66.3 ± 48.5 m from the urban boundary (median = 65, range: 10–125). Plasticity in habitat use has been previously described for cats. For example, in a recent study of GPS-tracked cats in Israel, roaming along roads, in buildings, and in open urban, natural, and agricultural areas was non-selective [63]. The same was observed in pet cats in Australia, which generally showed no preference for urban habitats or vegetated space [64]. This is in contrast to other studies describing cats as being more associated with human infrastructure than with natural areas [19,38,65].

Notwithstanding, a high level of conservation awareness is needed when pet cats reside near environmentally sensitive areas, as they might roam into these [38] and return prey at more than twice the rate of suburban cats [66]. This was not evidenced for fragments of native vegetation, though [60], which makes us think that cats might wander into green areas for reasons other than predation only. We are aware that GPS tracking is the more adequate method to explicitly assess habitat use in cats (see the solid studies with over 90 individuals by Bischof et al. [62] and Jensen et al. [67]). However, our study highlights the potential of animal-borne cameras to enable small-scale assessments that are not always feasible with GPS data [63], thereby contributing to our understanding of what cats actually do in different environments.

Different from hunting behaviors and preference for urban or more natural environmental settings, the cats’ exploration patterns were relatively uniform but deserve attention, as they were the most frequently displayed behaviors (considering the mean values, measured both as point events and for state event behaviors; Figure 3). Those behaviors have been less commonly described in behavioral studies of cats compared to the bolder and more relevant predation behaviors. However, if we better understand how exploration and hunting are related, we might better understand predation in cats. For example, exploration increases opportunities to acquire experience with prey, which, when gained during kittenhood, can later influence hunting preferences and skills [68]. Indeed, one of our detected trends showed that cats whose owners reported hunting exhibited higher levels of exploratory behaviors.

Finally, we found a trend that older cats were reported by their owners to hunt more, a result at odds with earlier studies reporting higher prey returns for younger cats [60,69]. Importantly, true predation rates as evidenced by video recording are significantly higher than owner-reported return rates [35]. In turn, whether a cat returns prey might depend on specific cat characteristics or its bond to the owner, thus leaving some uncertainty regarding this measurement of predation. In our study, for example, the reliability of owner observations on prey can be questioned, as rabbits and lizards reported by two participants are actually absent on the island [40]. Rabbits, though, might be temporarily present as escaped pets in the surroundings of Puerto Williams (Elke Schüttler, personal observation). There was also no correlation between owner-reported hunting activity and hunting-related behaviors in the camera footage; in fact, we could not video-record any live predation event, in contrast to findings from other studies (e.g., 40 successful captures in 23 out of 37 cats in New Zealand [70] and 39 in 16 out of 55 cats in the USA [35]). This makes us highlight some important limitations of our research. It would have been desirable to monitor more than 12 individuals and for longer time periods. However, few households (14% of 215) in Puerto Williams hold cats [42]; therefore, recruitment was limited. We also could not afford to buy technologically advanced cameras, such as the KittyCams produced by National Geographic, USA [35,36,70]; instead, we used commercially available pet cameras, which allowed recording of sessions up to ~3 h only, compared to the 10–12 h recording of KittyCams. This shortcoming might have reduced our chance to film the relatively rare predation events (stray cats spent on average 89.5% of their time inactive and only 0.9% hunting [71]). However, the commercially available cameras did their job and hence represent an affordable option for countries where conducting science is financially challenging.

## 5. Conclusions

In this explorative study, we have addressed a relatively new driver of cat–wildlife conflict, namely, the cat–owner relationship. Although we did not find a direct influence of this driver in the cat’s propensity to interact with wildlife, we highlight that owners reporting a closer relationship observed their cats disturbing wildlife more often. Hence, if owners pay more attention to their cats—as part of a strong relationship—they become more aware of their cats as predators of wildlife. Given the diversity of perceptions of hunting among cat owners, this does not necessarily mean that owners assume responsibility for their cats [50]. However, if behavioral change initiatives directed at cat owners address seemingly conflicting values, such as cat welfare and outdoor restriction, cat containment may be achieved gradually [50,54]. As cats largely behave individually, we recommend that cat owners carefully observe their cats, maybe even using commercial videocams, as in this study, to identify possible hunters and assume responsibility. This responsibility should start with night-time confinement [24,54,72], the use of collar-mounted bells or multicolored sleeves [73,74], and increased cat–owner interaction [30], such as play [22], as initial measures to promote coexistence between cats and wildlife.

## Figures and Tables

**Table 1:** Total effort of video-monitored cats on Navarino Island, southern Chile. One session refers to a visit to the owner with the charged videocam and fitting the cat with the device for a period of ~3 h. The monitoring period refers to the month and year when the sessions were conducted, total time monitored refers to the time the cat wore the device, and total time active is the time the camera recorded behavior.

Cat ID	Location	No. of Sessions	Monitoring Period	Total Time Monitored (hh:mm)	Total Time Active (hh:mm)
F01	Urban	4	October 2018	07:26	04:10
F02	Urban	3	October 2018	05:22	03:23
F03	Urban	5	December 2017/December 2018	16:11	06:28
F04	Urban	3	January 2019	7:17	02:26
F05	Rural	6	November/December 2017	12:35	05:29
F06	Urban	8	October/November 2017/December 2018	18:11	05:28
M01	Urban	3	November 2017	04:23	00:44
M02	Urban	3	April 2019	05:00	01:42
M03	Rural	5	October 2018/January 2019	07:29	06:19
M04	Urban	2	October 2018/February 2019	04:05	00:23
M05	Urban	4	December 2018/April 2019	05:18	02:41
M06	Urban	6	November/December 2017	13:07	11:20

**Table 2:** Overview of response and explanatory variables used in univariate non-parametric statistical tests to determine factors influencing the cat outdoor behavior of 12 free-ranging cats on Navarino Island, southern Chile, based on a husbandry-related questionnaire given to owners, behavior recorded by videocams, and the CORS questionnaire. The values in parenthesis in the data type column indicate the empirical data range.

Variable Classification	Variable Description	Data Type (Range)
	Response variables	
Behavior	Percentage occurrences of hunting-related behaviors (foraging, hunting, stalking, stare—hunting)	Continuous (0–2.5%)
Behavior	Percentage occurrences of exploratory-related behaviors (exploring, interaction—objects, interaction—social, watching)	Continuous (22.6–78.5%)
Behavior	Percentage time spent in nature (i.e., shrubland, forest)	Continuous (0–48.9%)
Owner observation	Cat has been observed hunting an animal/bird in past year (i.e., dead prey brought home)	Categorical (no/yes)
Owner observation	Cat has been observed harassing an animal/bird in past year (i.e., prey was chased, but not killed)	Categorical (no/yes)
	Explanatory variables	
Husbandry	Parasite treatment up to date (<3 months)	Categorical (no/yes)
Husbandry	Number of households cat receives food	Numerical (1–2)
Husbandry	Cat’s role for owner	Categorical (company/rodent control)
Husbandry	Precedents of having gone lost	Categorical (no/yes)
Husbandry	Restriction pattern	Categorical (no/yes)
Intrinsic	Age	Continuous (1–13)
Intrinsic	Sex	Categorical (female/male)
Owner relationship	Cat–Owner Relationship Scale (sum of Interaction and Emotion subscales, Cost subscale excluded due to missing internal consistency)	Continuous (5.8–9.5)

## Data Availability

The data presented in this study are included in an Excel file in the Supplementary Materials (Table S2). Further inquiries can be directed to the corresponding author (E.S.). Complete video data are unavailable due to ethical restrictions (anonymous participation of cat owners).

## Supplementary Materials

The following supporting information can be downloaded at https://www.mdpi.com/article/10.3390/ani16172777/s1: Table S1: Translated (to Spanish) slightly modified version of the Cat–Owner Relationship Scale (CORS) developed by Howell et al. [31]; Table S2: Excel file of overall data; Video S1: Video excerpt of M06 sniffing a dead bird; Video S2: Video excerpt of F06 exploring the forest; Video S3: Video excerpt of M05 observing a bird in town.

## Abbreviations

The following abbreviations are used in this manuscript:

CORS	Cat–Owner Relationship Scale
PCA	Principal Component Analysis

## Appendix A

**Table A1:** Questionnaire to assess demographic and husbandry data of 12 free-ranging cats on Navarino Island, southern Chile, as well as data on the background of cat owners. The questionnaire was adapted from previous research in the study area [42].

Question
Cat data
1. Please indicate the following information about your cat: Age, sex, origin, how cat was obtained, why cat is owned.
2. If your cat is female and has given birth, please indicate the following information on its kittens: date of birth, no. of kittens, no. of dead kittens, no. of eliminated kittens, no. of adopted/given away (where to?).
3. Has your cat gone missing during the past year? If yes, how many days, what do you think why?
4. Is your cat neutered?
5. Is your cat receiving antiparasitic treatment? If yes, when was the last time?
6. Did you vaccinate your cat against rabies? If yes, when was the last time?
7. What did your cat eat yesterday?
8. What do you feed your cat typically?
9. In how many households does your cat receive food?
10. Do you restrict your cat’s movement? If yes, at day and/or at night?
11. What do you do with your cat when you have to travel?
12. What would you need to improve the care of your cat?
13. In the past year, has your cat returned home with an animal/bird? If yes, which?
14. In the past year, have you ever seen your cat hunting another animal? If yes, when and which?
Cat owner data
15. Gender
16. Age
17. Highest completed education level: a. none, b. elementary school, c. high school, d. technical training, e. university
18. How much time (months/years) have you lived in Cape Horn County?
19. How many people live in your household? No. of adults, no. of children.
20. How many pets in total live in your household?

**Table A2:** Ethogram of selected behaviors among 13 behavioral categories described by Stanton et al. [43] based on the catcam ethogram from Huck & Watson [44] to analyze the outdoor behavior of 12 free-ranging cats on Navarino Island, southern Chile. Note that behavioral categories should not be considered exhaustive [45]. The behavior purr was extracted from [45], the interaction behaviors (social/objects) were own classifications. Short point event behaviors were recorded as frequencies of occurrence, while longer state event behaviors were recorded as both frequencies of occurrence and the time the behavior persisted (measured in s).

Behavior	Category	Description	Sampling
Approaching	Agonistic, locomotion	Cat moves toward (modifier) while looking at it.	Point event
Clawing	Active, maintenance, marking, reproductive	Cat drags front claws along an object or surface, likely leaving visual marks behind.	State event
Climbing	Locomotion	Cat ascends and/or descends an object or structure.	State event
Drinking	Active, feeding, maintenance	Cat ingests water (or other liquids) by lapping up with the tongue.	State event
Eating	Active, feeding, maintenance	Cat ingests food (or other edible substances) by chewing with the teeth and swallowing.	State event
Exploring	Active, exploratory	Cat moves around attentively while sniffing the ground and/or objects.	State event
Foraging	Active, feeding	Cat searches for food or other edible substances.	Point event
Grooming	Active, calm, fear, inactive, maintenance	Cat cleans itself by licking, scratching, biting or chewing the fur on its body. May also include the licking of a front paw and wiping it over one’s head.	State event
Hunting	Active, feeding	Cat actively pursues live prey. Includes movements such as crouching, stalking, or any other species-specific behavior.	Point event
Interacting (objects)	Active, exploratory	Cat shows interest in an inanimate object, touching it with its paws or playing with it.	Point event
Interacting (social)	Active, exploratory	Cat seeks attention or shares a specific area with another individual (dog, cat, or human) with no sign of aggression.	State event
Jumping	Locomotion	Cat leaps from one point to another, either vertically or horizontally.	Point event
Lying	Calm, inactive	Cat’s body is on the ground in a horizontal position, including on its side, back, belly, or curled in a circular formation.	State event
Purring	Calm, vocalizations	Low, continuous rhythmical tone produced during respiration while the cat’s mouth is closed. Creates a murmuring sound.	State event
Resting alert	Active, calm, inactive	Sitting: Cat is in an upright position, with the hind legs flexed and resting on the ground, while front legs are extended and straight. Standing: Cat is in an upright position and immobile, with all four paws on the ground and legs extended, supporting the body.	State event
Rolling	Active, affiliative, agonistic, marking	While lying on the ground, cat rotates its body from one side to another. During the roll, the back is rubbed against ground, the belly is exposed and all paws are in the air. Cat may continue rolling repeatedly from side to side.	Point event
Running	Locomotion	Forward locomotion in a rapid gait, which is faster than walking or trotting.	State event
Scratching	Active, calm	Cat scratches its body using the claws of its hind feet.	Point event
Sleeping	Inactive	Cat is lying on the ground with its head down and eyes closed, performing minimal head or leg movement, and is not easily disturbed.	State event
Stalking	Feeding, locomotion	Slow, forward locomotion in a crouched position directed toward (modifier), with head kept low and eyes focused on (modifier).	State event
Stare—hunting	Active, feeding	Cat gazes fixedly at (modifiers) and is not easily distracted.	Point event
Stare—social	Agonistic	Cat gazes fixedly at another cat and is not easily distracted.	State event
Stretching	Active, calm, inactive	Cat extends its forelegs while curving its backs inwards.	Point event
Touching noses	Affiliative	Two cats sniff at and touch each other with their noses.	Point event
Vocalizing	Active, vocalizations	Cat produces sounds or calls, originating from the throat and mouth.	Point event
Walking	Locomotion	Forward locomotion at a slow gait.	State event
Watching	Exploratory	Cat observes a specific stimulus (or modifier).	State event
