# Supplementary material for: Is the Cat–Owner Relationship Related to Cat–Wildlife Conflicts?
Source: Animals (Basel). 2026 Sep 3;16(17):2777. doi: 10.3390/ani16172777 (PMC13565001; doi:10.3390/ani16172777)
Supplement: Supplementary file 1 [file animals-16-02777-s001.zip › Table S1.pdf]

Table S1

Translated slightly modified version of the Cat-Owner Relationship Scale (CORS) developed by Howell et al. (2017) to assess the strength of the relationship between cats and their owners (from the owner´s perspective) among 12 cat-owner dyads in southern Chile. Question 31 (How often do you buy your pet presents?) of the original questionnaire was omitted due to the limited offer of commercial pet articles in Puerto Williams during the study.

**ID**	**Pregunta**	**Opciones de respuesta**				
1	¿Qué tan difícil es cuidar a su gato?	Muy difícil	Difícil	Ni difícil, ni fácil	Fácil	Muy fácil
2	Mi gato me da una razón para levantarme en las mañanas	Muy de acuerdo	De acuerdo	Ni de acuerdo, ni en desacuerdo	En desacuerdo	Muy de acuerdo
3	Hay aspectos importantes de ser dueño de un gato que no me agradan	Muy de acuerdo	De acuerdo	Ni de acuerdo, ni en desacuerdo	En desacuerdo	Muy en desacuerdo
4	¿Con qué frecuencia le da besos a su gato?	Al menos una vez al día	Una vez cada cuántos días	Una vez a la semana	Una vez al mes	Nunca
5	Me gustaría que mi gato y yo nunca estuviéramos separados	Muy de acuerdo	De acuerdo	Ni de acuerdo, ni en desacuerdo	En desacuerdo	Muy en desacuerdo
6	Mi gato genera demasiado desorden	Muy de acuerdo	De acuerdo	Ni de acuerdo, ni en desacuerdo	En desacuerdo	Muy en desacuerdo
7	¿Qué tan seguido juega con su gato?	Al menos una vez al día	Una vez cada cuántos días	Una vez a la semana	Una vez al mes	Nunca
8	Me molesta que mi gato no me permita hacer cosas que antes me gustaban hacer	Muy de acuerdo	De acuerdo	Ni de acuerdo, ni en desacuerdo	En desacuerdo	Muy en desacuerdo
9	¿Cuántas veces simplemente disfruta observando a su gato?	Al menos, una vez a la semana	Una vez a la semana	Una vez al mes	Algunas veces al año	Nunca
10	Me molesta que a veces tenga que cambiar mis planes debido a mi gato	Muy de acuerdo	De acuerdo	Ni de acuerdo, ni en desacuerdo	En desacuerdo	Muy en desacuerdo
11	Cuidar a mi gato cuesta demasiado dinero	Muy de acuerdo	De acuerdo	Ni de acuerdo, ni en desacuerdo	En desacuerdo	Muy en desacuerdo
12	¿Qué tan seguido compra regalos para su gato?	Una vez a la semana	Cada dos semanas	Una vez al mes	Algunas veces al año	Nunca
13	¿Qué tan a menudo le dice a su gato cosas que no le cuenta a nadie más?	Una vez al día	Una vez a la semana	Una vez al mes	Una vez al año	Nunca
14	¿Qué tan a menudo siente que cuidar a su gato es una tarea difícil?	Una vez al día	Una vez a la semana	Una vez al mes	Una vez al año	Nunca

15	¿Con qué frecuencia habla a su gato?	Al menos, una vez al día	Una vez cada cuántos días	Una vez a la semana	Una vez al mes	Nunca
16	¿Con qué frecuencia su gato no le permite hacer las cosas que quiere?	Una vez al día	Una vez a la semana	Una vez al mes	Una vez al año	Nunca
17	Me gustaría estar cerca de mi gato todo el tiempo	Muy de acuerdo	De acuerdo	Ni de acuerdo, ni en desacuerdo	En desacuerdo	Muy en desacuerdo
18	Si todos los demás me dejarían, mi gato aún seguiría allí para mí	Muy de acuerdo	De acuerdo	Ni de acuerdo, ni en desacuerdo	En desacuerdo	Muy en desacuerdo
19	¿Qué tan a menudo siente que tener un gato es más un problema que un beneficio?	Una vez al día	Una vez a la semana	Una vez al mes	Una vez al año	Nunca
20	Mi gato me ayuda a superar tiempos difíciles	Muy de acuerdo	De acuerdo	Ni de acuerdo, ni en desacuerdo	En desacuerdo	Muy en desacuerdo
21	¿Con qué frecuencia regalonea a su gato?	Al menos, una vez al día	Una vez cada cuántos días	Una vez a la semana	Una vez al mes	Nunca
22	Mi gato me acompaña constantemente	Muy de acuerdo	De acuerdo	Ni de acuerdo, ni en desacuerdo	En desacuerdo	Muy en desacuerdo
23	¿Con qué frecuencia su gato está con usted mientras se relaja? (por ejemplo: mirando TV)	Al menos, una vez al día	Una vez cada cuántos días	Una vez a la semana	Una vez al mes	Nunca
24	Mi gato está allí cuando necesito consuelo	Muy de acuerdo	De acuerdo	Ni de acuerdo, ni en desacuerdo	En desacuerdo	Muy en desacuerdo
25	¿Qué tan doloroso cree que será para usted cuando su gato muera?	Extremadamente doloroso	Muy doloroso	Doloroso	Poco doloroso	Para nada doloroso
26	¿Con qué frecuencia acaricia a su gato?	Al menos, una vez al día	Una vez cada cuántos días	Una vez a la semana	Una vez al mes	Nunca
27	¿Con qué frecuencia lleva a su gato a visitar a otras personas?	Una vez a la semana	Cada dos semanas	Una vez al mes	Algunas veces al año	Nunca
28	¿Con qué frecuencia le da a su gato premios de comida (aparte de su comida regular)?	Al menos, una vez al día	Una vez cada cuántos días	Una vez a la semana	Una vez al mes	Nunca
29	¿Con qué frecuencia lleva a su gato en su auto?	Al menos, una vez al día	Una vez cada cuántos días	Una vez a la semana	Una vez al mes	Nunca

30	¿Con qué frecuencia abraza a su gato?	Al menos, una vez al día	Una vez cada cuántos días	Una vez a la semana	Una vez al mes	Nunca
31	¿Con qué frecuencia peina a su gato?	Al menos, una vez al día	Una vez cada cuántos días	Una vez a la semana	Una vez al mes	Nunca
32	Mi gato está constantemente pendiente de mí	Muy de acuerdo	De acuerdo	Ni de acuerdo, ni en desacuerdo	En desacuerdo	Muy en desacuerdo

References

Howell, T.J.; Bowen, J.; Fatjó, J.; Calvo, P.; Holloway, A.; Bennett, P.C. Development of the cat-owner relationship scale (CORS). *Behav. Process*., 2017, *141*, 305–315. https://doi.org/10.1016/j.beproc.2017.02.024
